# The Book World of Medicine and Science

**Published:** 1896-01-25

**Authors:** 


### THE BOOK WORLD OF MEDICINE AND SCIENCE.

THE DISEASES OF CHILDREN'S TEETH: THEIR PREVENTION AND TREATMENT. By R. DENISON PEDLEY, M.R.C.S., L.D.S.Eng., F.R.C.S.E. (Published by J. P. Segg and Co., pp. 268, with 99 illustrations. Price 7s. 6d.)

This book is specially intended for the use of practitioners and students of medicine to whom Mr. Pedley eloquently appeals to devote more attention to this branch of surgery, and the soundness of this advice is unquestionable, and may, we hope, be followed by those who propose to become general practitioners in the country. Mr. Pedley's manual is clearly and pleasantly written, and constitutes a fairly exhaustive treatise on diseases of teeth and their treatment, beginning with an account of their structure and eruption, then dealing with the various diseases and irregularities to which they are subject, and finally discoursing on treatment. The dangers to which the teeth are exposed may well impress the non-dental reader with the precariousness of these bony structures. Then the author says "weak acids are capable of dissolving out the lime-salts, leaving behind a gelatinous matrix, and such acids are constantly found in the mouth." And again, "the mouth is an ideal chamber with all the conditions favourable for the action and propagation of micro-organisms, and the particles of food left upon or in the neighbourhood of the teeth, are the natural essential for cultivation." It is now well recognised that the production of caries in teeth depends entirely on the presence of micro-organisms in the mouth; thirty species of them have been isolated and cultivated, twelve of them being characterised by the formation of lactic acid. Mr. Pedley considers that modern teeth are far more liable to caries than were those of the ancients, and since there is a tendency to suppression of some members of the dental series, and to the jaw becoming smaller owing to the great improvement in preparing food, one feels tempted to wish that in the far-off future the whole series may be suppressed, and that men, women, and children may be saved from the torture and worry of unsatisfactory structures. Till this happy state of things arrives we can only hope that many general practitioners may be found to study this useful volume, and fit themselves to relieve some of the terrible pain connected with the possession of teeth.

THE TALLERMAN-SHEFFIELD PATENT LOCALISED HOT-AIR BATH. (London: Ballière, Tindall, and Cox. 1895.)

This is a book apparently without an author, and is, in fact, a mere advertisement of a hot-air bath and an institute for the application of the same. No details are given, however, as to the nature of the apparatus beyond the statement that it consists of a copper chamber, and that "the secret lies in an ingenious arrangement for keeping the air really dry." The secret in a patent! Some interesting cases as to the efficacy of the local application of hot air in gout, rheumatism, and contractures of joints are given over the signatures of various medical authorities, and a clinical lecture delivered at St. Bartholomew's Hospital by Mr. Alfred Willett is quoted by permission. Anyone who is familiar with the results obtained at various hydropathic establishments knows well enough the benefit which often follows the application of heat to painful and stiff joints, and it is sufficiently well known that by the application of dry heat there is superadded to these advantages those which attend free diaphoresis. The essential thing in any apparatus is that means shall be employed to prevent the accumulation of the vapour which arises from the evaporation of the perspiration, and in this the apparatus here mentioned seems to be successful. Some of the cases recorded are very striking.

